# BAZ1B is dispensable for H2AX phosphorylation on Tyrosine 142 during spermatogenesis

**DOI:** 10.1242/bio.011734

**Published:** 2015-05-15

**Authors:** Tyler J. Broering, Yuan-Liang Wang, Ram Naresh Pandey, Rashmi S. Hegde, Shao-Chun Wang, Satoshi H. Namekawa

**Affiliations:** 1 Division of Reproductive Sciences, Cincinnati Children's Hospital Medical Center, Cincinnati, OH 45229, USA; 2 Division of Developmental Biology, Perinatal Institute, Cincinnati Children's Hospital Medical Center, Cincinnati, OH 45229, USA; 3 Department of Pediatrics, University of Cincinnati College of Medicine, Cincinnati, OH 45267, USA; 4 Department of Cancer Biology, University of Cincinnati College of Medicine, Cincinnati, OH 45267, USA

**Keywords:** Meiosis, Sex chromosomes, Spermatogenesis

## Abstract

Meiosis is precisely regulated by the factors involved in DNA damage response in somatic cells. Among them, phosphorylation of H2AX on Serine 139 (γH2AX) is an essential signal for the silencing of unsynapsed sex chromosomes during male meiosis. However, it remains unknown how adjacent H2AX phosphorylation on Tyrosine 142 (pTyr142) is regulated in meiosis. Here we investigate the meiotic functions of BAZ1B (WSTF), the only known Tyr142 kinase in somatic cells, using mice possessing a conditional deletion of BAZ1B. Although BAZ1B deletion causes ectopic γH2AX signals on synapsed autosomes during the early pachytene stage, BAZ1B is dispensable for fertility and critical events during spermatogenesis. BAZ1B deletion does not alter events on unsynapsed axes and pericentric heterochromatin formation. Furthermore, BAZ1B is dispensable for localization of the ATP-dependent chromatin remodeling protein SMARCA5 (SNF2h) during spermatogenesis despite the complex formation between BAZ1B and SMARCA5, known as the WICH complex, in somatic cells. Notably, pTyr142 is regulated independently of BAZ1B and is dephosphorylated on the sex chromosomes during meiosis in contrast with the presence of adjacent γH2AX. Dephosphorylation of pTyr142 is regulated by MDC1, a binding partner of γH2AX. These results reveal the distinct regulation of two adjacent phosphorylation sites of H2AX during meiosis, and suggest that another kinase mediates Tyr142 phosphorylation.

## INTRODUCTION

Germ cells undergo extensive chromatin changes to produce condensed haploid spermatozoa during spermatogenesis. The central event in spermatogenesis is meiosis, in which genetic materials are exchanged between parental chromosomes to confer genetic diversity in offspring. During meiosis, homologous chromosomes align, synapse, and exchange genetic material via meiotic recombination, and segregate into haploid gametes. Because of the necessity to cope with DNA breaks during meiotic recombination, the factors involved in recombination and DNA repair in somatic cells have essential functions in mammalian male meiosis (Baudat et al., 2013; Handel and Schimenti, 2010). In addition to meiotic recombination, the synaptic status of meiotic chromosomes is monitored by the DNA damage response (DDR) factors, which also function in somatic DNA repair. During the pachytene stage, DDR factors accumulate on unsynapsed chromatin that is then transcriptionally silenced (Baarends et al., 2005; Turner et al., 2005). In normal male meiosis, unsynapsed chromatin is restricted to sex chromosomes at the onset of meiotic sex chromosome inactivation (MSCI), which is an essential step in meiotic progression and male fertility (Ichijima et al., 2012; Turner, 2007). Due to the essential roles of DDR/DNA repair factors during meiosis, germ cells must coordinate chromatin remodeling and DDR/DNA repair events during the normal developmental process.

Phosphorylated H2AX at Ser139 (γH2AX) mediates somatic DDR (Bassing et al., 2002; Celeste et al., 2002) and has an essential function in the male germline. H2AX knockout mice lack MSCI (Fernandez-Capetillo et al., 2003); MDC1, a binding partner of γH2AX (Goldberg et al., 2003; Lou et al., 2003; Stewart et al., 2003), is required for the spreading of γH2AX to the chromosome-wide domain and initiation of MSCI (Ichijima et al., 2011). It remains elusive how the γH2AX domain is regulated in relation to dynamic chromatin remodeling in germ cells. One possible factor for such a function is BAZ1B (bromodomain adjacent to zinc finger domain, 1b; also known as WSTF: Williams syndrome transcription factor), a chromatin-remodeling factor and also a tyrosine-protein kinase that mediates Tyr142 phosphorylation of H2AX (pTyr142) in somatic cells (Xiao et al., 2009). Knockdown of *Baz1b* diminished Tyr142 phosphorylation and caused maintenance defects of γH2AX (Xiao et al., 2009). Subsequent studies demonstrated that dephosphorylation of pTyr142 is mediated by the eyes absent (EYA) family of protein phosphatases (Cook et al., 2009; Krishnan et al., 2009), and that pTyr142 regulation is critical for the balance between DNA repair and apoptosis (Cook et al., 2009). However, the *in vivo* role of BAZ1B in the germline remains unknown.

As a chromatin remodeling factor, BAZ1B binds to SMARCA5 (also known as SNF2H), a mouse homolog of imitation switch (ISWI), to form the ATP-dependent chromatin remodeling complex WICH, which regulates replication foci on heterochromatin (Bozhenok et al., 2002). BAZ1B and SMARCA5 also form the complex WICH-B with several other proteins in transcription (Cavellan et al., 2006). Although the *in vivo* function of BAZ1B and SMARCA5 remains unknown, an *in vivo* screening of epigenetic modifiers using ENU mutagenesis identified BAZ1B and SMARCA5 as critical epigenetic modifiers (Ashe et al., 2008; Chong et al., 2007). These studies identified *Momme* (*Modifiers of murine metastable epialleles*) mutants, among which *MommeD4* was the *Smarca5* mutant and *MommeD10* was the *Baz1b* mutant. Notably, in the germline, haploinsufficiency of these mutants was associated with paternal epigenetic effects in the next generation (Ashe et al., 2008; Chong et al., 2007). These results suggest that the WICH complex has a critical function in the germline.

To address the functions of BAZ1B in the germline, we generated germline-specific conditional-knockout mutants of *Baz1b* (*Baz1b*cKO). Unexpectedly, spermatogenesis was largely unaffected in *Baz1b*cKO mice despite ectopic γH2AX signaling in the early pachytene stage. Likewise, localization of pTyr142 and SMARCA5 was undisturbed. We found that pTyr142 is dephosphorylated on the sex chromosomes during wild-type meiosis in an MDC1-dependent manner, although we did not determine the mechanism by which Tyr142 of H2AX is phosphorylated during spermatogenesis. These results reveal that MDC1 coordinates both dephosphorylation of pTyr142 and phosphorylation of Ser139 of H2AX on the sex chromosomes during meiosis, and suggest the possibility that another kinase mediates pTyr142.

## RESULTS

### BAZ1B is not required for male fertility

**Fig. 1.**
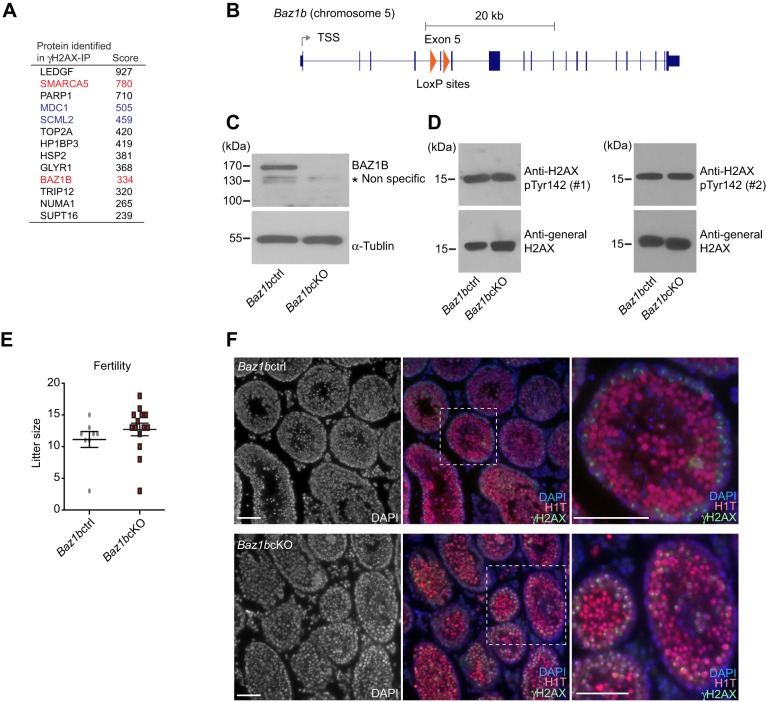
**BAZ1B is dispensable for male fertility.** (A) γH2AX-associated proteins identified by mass spectrometry (Hasegawa et al., 2015). Functions of MDC1 and SCML2 during meiosis were previously reported (shown in blue, Ichijima et al., 2011, Hasegawa et al., 2015). SMARCA5 and BAZ1B are focuses of this study (shown in red). (B) Structure of *Baz1b* gene. The regions inserted with loxP sites are shown in red triangles. (C) Western blotting with anti-BAZ1B antibody. α-tublin was used for the loading control. (D) Western blotting with two independent anti-BAZ1B antibodies. The antibody from Millipore was used for the left panel (#1), and the antibody from Abcam was used for the right panel (#2). α-tublin was used for the loading control. (E) Fertility test: Litter sizes (±s.e.m.) fathered by the *Baz1b*cKO mice or *Baz1b*cKO mice with wild-type CD1 females are shown. A total of 7 *Baz1b*cKO males and 3 *Baz1b*ctrl males were used for breeding. Each male was mated with 1–3 wild-type CD1 females at the same time in the same cage. Total number of pregnancies examined: 17 for the *Baz1b*cKO mice and 9 for *Baz1b*ctrl mice. (F) Immunostaining of testicular sections stained with H1T and γH2AX. Scale bars, 100 µm. The areas shown with white dotted squares are magnified in the right panels.

Since the regulation of H2AX phosphorylation (Ser 139 and Tyr 142) is crucial to somatic DDR (Cook et al., 2009), we initially sought to examine the functional relationship between these two sites in the germline where DDR events occur in the context of normal developmental processes. To this end, we focused on BAZ1B, the sole known enzyme that mediates Tyr142 phosphorylation of H2AX (pTyr142) in somatic cells (Xiao et al., 2009). In our recent study, we detected two components of the WICH complex (SMARCA5 and BAZ1B) in the mass spectrometry analyses of γH2AX-containing nucleosomes purified from adult testes (Fig. 1A) (Hasegawa et al., 2015), suggesting that BAZ1B may work in the context of γH2AX. Based on the reduced viability of homozygous mice harboring a point mutation of *Baz1b* (*MommeD10*) (Ashe et al., 2008), we generated the conditional deletion of *Baz1b* in the germline to test the function of BAZ1B during spermatogenesis. In these mice, termed *Baz1b* conditional knockout (*Baz1b*cKO), *Baz1b* exon 5 was excised using *Ddx4*-Cre, which expresses Cre proteins specifically in the germline starting from embryonic day 15 (Fig. 1B) (Gallardo et al., 2007). We predicted that removal of exon 5 would cause premature stop codon and nonsense-mediated mRNA decay of *Baz1b*. Consistent with this prediction, the BAZ1B protein was not detected in the crude extract of *Baz1b*cKO adult testes (Fig. 1C). In spite of the depletion of BAZ1B proteins in *Baz1b*cKO, there was no change in the level of pTyr142 between *Baz1b*cKO and their control littermates (*Baz1b F/+Ddx4-cre* controls, termed *Baz1b*ctrl). By using two independent anti-pTyr142 antibodies, we confirmed that the total amounts of pTyr142 in testicular extracts of adult testes are consistent between *Baz1b*ctrl and *Baz1b*cKO (Fig. 1D). Because pTyr142 was maintained through mitotic proliferation after the depletion of BAZ1B in embryonic germ cells, these results suggest that, contrary to our prediction, BAZ1B is not required for Tyr142 phosphorylation in male germ cells.

Furthermore, to our surprise, *Baz1b*cKO males and females were both fertile (Fig. 1E and data not shown). To test for fertility, *Baz1b*cKO males were mated with CD-1 females. Litter sizes fathered by the *Baz1b*cKO mice were similar in number to those of WT males (Fig. 1E). Although mice heterozygous for *Baz1b* knockout alleles were viable, we obtained only one global knockout mouse, a male, from heterozygous breeding pairs over a two-year period. Thereby, we characterize only *Baz1b*cKO mice hereafter. It should be noted that the body size of the homozygous mutant mouse was small in comparison to the control littermates (data not shown). Small body size and reduced viability of homozygous mutants are consistent with *MommeD10* (Ashe et al., 2008). Consistent with the fertility of *Baz1b*cKO mice, the morphology of testicular tubules was apparently normal, and germ cells reached the meiotic stages, as judged by accumulation of γH2AX and H1T, a testis-specific linker histone that is enriched after the mid-pachytene stage (Fig. 1F).

### Increased apoptosis in early pachytene spermatocytes of *Baz1b*cKO male mice

**Fig. 2.**
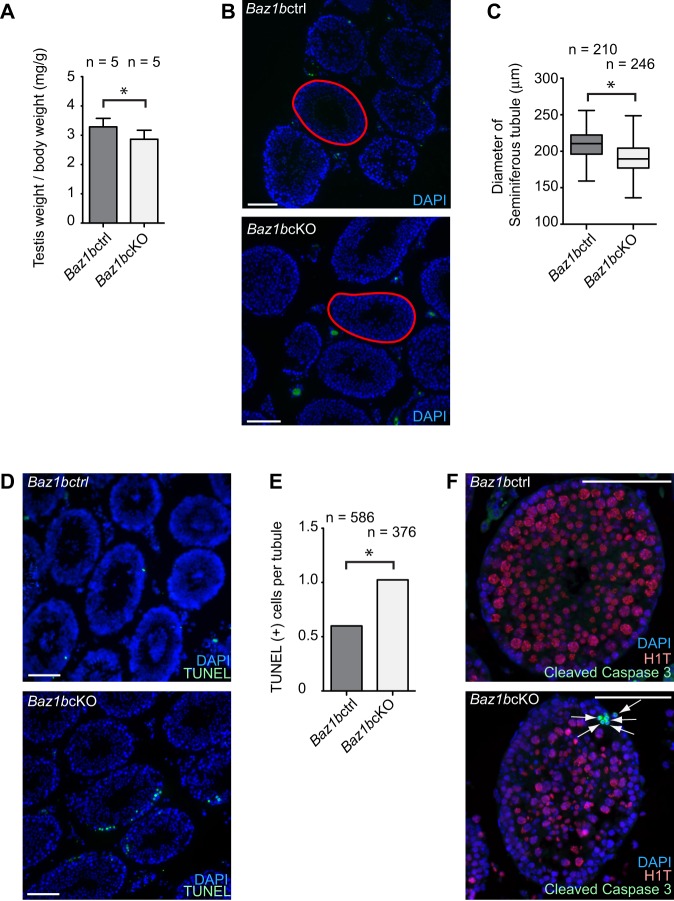
***Baz1b* conditional deletion causes germ cell loss at the early pachytene stage.** (A) Testis weights/body weight (mg/g; ±s.d.) of *Baz1b*ctrl or *Baz1b*cKO mice at 18 weeks after birth. Five independent mice were analyzed for *Baz1b*ctrl or *Baz1b*cKO mice. (B) Representative photo of the testes of *Baz1b*ctrl or *Baz1b*cKO mice. An example of the area measurement of a tubule was shown in red lines. (C) Diameter of seminiferous tubule areas (µm) at 11 weeks after birth. Two independent mice were analyzed for *Baz1b*ctrl or *Baz1b*cKO mice. The central bar is the median, the boxes encompass 50% of data points and the top and bottom bars indicate 90% of data points. (D) TUNEL assays of testicular sections. (E) Scoring of the average number of TUNEL-positive cells per germ cell-containing tubule. Total numbers of tubules analyzed at 11 weeks from two independent *Baz1b*ctrl or *Baz1b*cKO mice are shown. (F) Immunostaining of Cleaved Caspase 3 and H1T in adult testes. Apoptotic cells are shown with arrows. Scale bars, 100 µm. *P<0.05, unpaired *t*-test.

To determine whether BAZ1B has any potential roles in the germline, we further characterized the mutant testes in detail. The average testicular weight was slightly smaller in *Baz1b*cKO compared to that of littermate controls [average ratio of testis weight (mg) per body weight (g): 2.89 in *Baz1b*cKO, 3.25 in *Baz1b*ctrl, Fig. 2A]. In accordance with smaller testes, the diameter of each seminiferous tubule was also smaller in *Baz1b*cKO (average diameter of each seminiferous tubule: 191.0 μm in *Baz1b*cKO, 206.3 μm in *Baz1b*ctrl, Fig. 2B,C). Because the smaller tubules suggest the possibility of germ cell loss by apoptosis during differentiation of spermatogenic cells, we performed apoptosis assays. Using TUNEL staining to detect apoptotic cells in testicular sections, we determined that *Baz1b*cKO mice exhibited a higher number of apoptotic cells per tubule than that of littermate controls (Fig. 2D,E). To determine the stage at which increased apoptosis occurs in *Baz1b*cKO mice, we stained testicular sections with antibodies against cleaved Caspase 3, a marker of apoptotic cells (Gown and Willingham, 2002), together with the stage marker H1T. In *Baz1b*cKO, cleaved Caspase 3-positive cells located at the periphery of tubules and were devoid of H1T staining (Fig. 2F) indicating that apoptosis is increased prior to the mid-pachytene stage in *Baz1b*cKO.

### Ectopic γH2AX signaling in early pachytene spermatocytes of *Baz1b*cKO mice

**Fig. 3.**
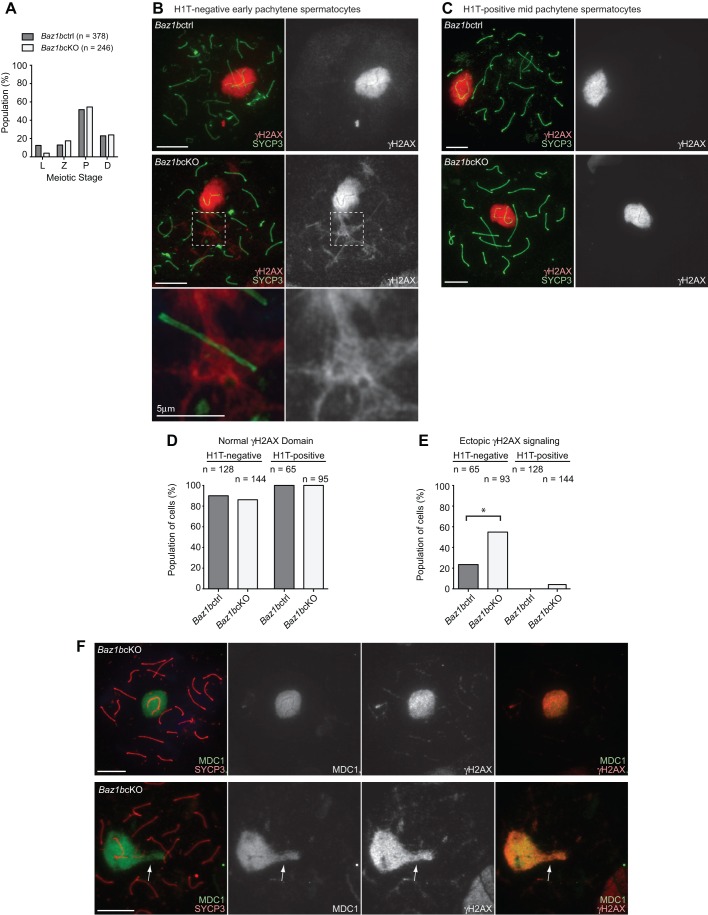
**BAZ1B regulates γH2AX-MDC1 signaling in early pachytene spermatocytes.** (A) The percentage of each stage of meiotic prophase as judged by SYCP3 immunostaining from two independent *Baz1b*ctrl or *Baz1b*cKO mice. (B,C) Immunostaining of chromosome spreads with γH2AX and SYCP3. Scale bars, 10 µm unless otherwise indicated. The areas shown with white dotted squares are magnified in the bottom panels. (D) Scoring of population of cells with normal γH2AX domain on the sex chromosomes from two independent *Baz1b*ctrl or *Baz1b*cKO mice. (E) Scoring of population of cells with ectopic γH2AX signaling on synapsed autosomal regions from two independent *Baz1b*ctrl or *Baz1b*cKO mice. (F) Immunostaining of chromosome spreads with MDC1 and SYCP3. Arrows indicate ectopic γH2AX-MDC1 signaling on synapsed autosomal regions. Scale bars, 10 µm. *P<0.05, unpaired *t*-test.

To further investigate the cause of early pachytene loss in *Baz1b*cKO, we looked into the detail of meiotic phenotypes of *Baz1b*cKO using meiotic chromosome spreads. Although we examined meiotic stage progression by scoring the population of each stage of meiotic prophase judged by SYCP3 staining, all substages of meiotic prophase were observed and meiotic progression was not significantly altered in *Baz1b*cKO (Fig. 3A). However, we found a notable abnormality in the early pachytene stage. In normal meiosis of early pachytene spermatocytes, which is judged by the absence of H1T, γH2AX spreads onto the chromosome-wide domain of the sex chromosomes at the onset of MSCI and γH2AX signals are diminished on synapsed autosome regions, as shown in *Baz1b*ctrl (Fig. 3B). In *Baz1b*cKO, although γH2AX domain formation on the sex chromosome is normal compared to that of *Baz1b*ctrl (Fig. 3B,D), ectopic γH2AX on autosome regions was more frequently observed than that of *Baz1b*ctrl (Fig. 3B,E). However, chromosome synapsis occurred normally at the sites of ectopic γH2AX signals on the autosomal regions (magnified panel in Fig. 3B). These results suggest that the ectopic γH2AX domain is caused by abnormal DDR signaling on synapsed regions in *Baz1b*cKO rather than ectopic silencing of unsynapsed autosomes. In mid pachytene spermatocytes, judged by the presence of H1T, we did not observe frequent ectopic γH2AX signals on the autosome regions (Fig. 3C,E). Therefore, ectopic γH2AX signals on the autosomal regions are specific to the early pachytene stage and may be associated with early pachytene apoptosis in *Baz1b*cKO.

To further characterize the ectopic γH2AX signals in *Baz1b*cKO, we tested the localization of MDC1, an essential factor for the amplification of γH2AX from unsynapsed axes to the chromosome-wide domain of sex chromosomes (Ichijima et al., 2011). MDC1 localized with the normal γH2AX domain on the sex chromosomes both in *Baz1bctrl* and *Baz1b*cKO and also accumulated on ectopic γH2AX signals on the autosomes region in *Baz1b*cKO (Fig. 3F), suggesting that MDC1 is involved in the ectopic γH2AX signals in *Baz1b*cKO.

### BAZ1B does not regulate BRCA1, ATR, or TOPBP1 on unsynapsed axes

**Fig. 4.**
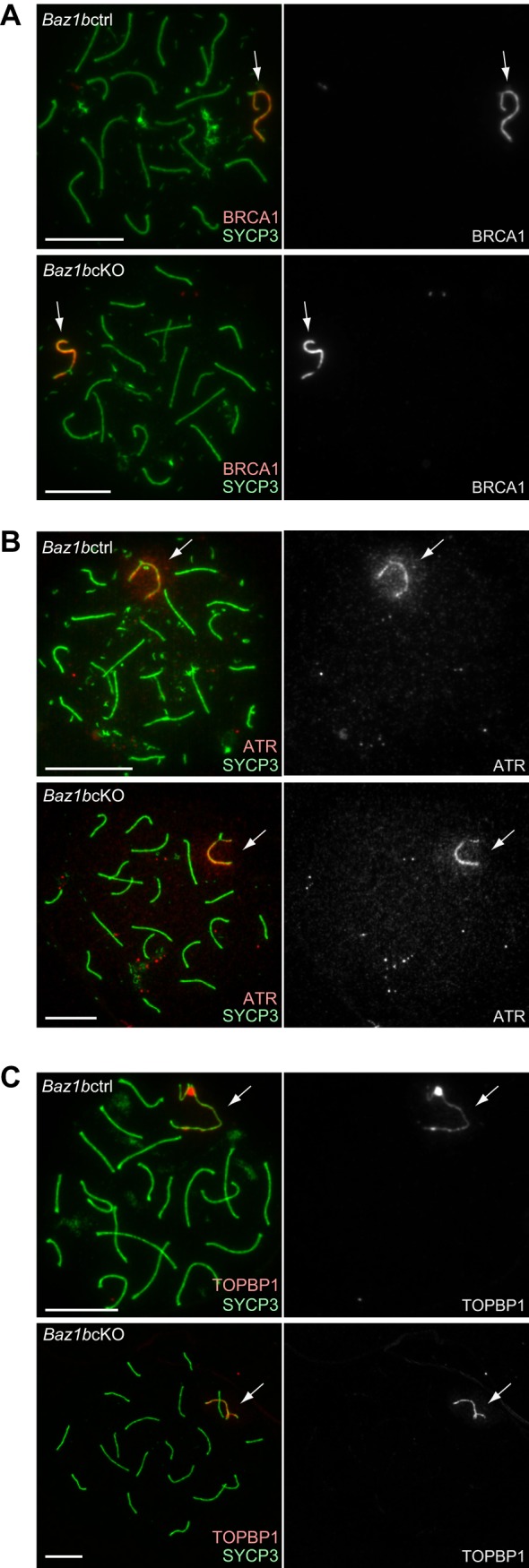
**BAZ1B does not regulate DNA damage signaling on unsynapsed axes.** Immunostaining of early pachytene spermatocytes using chromosome spreads with antibodies against SYCP3 and (A) BRCA1, (B) ATR and (C) TOPBP1. Scale bars: 10 µm. Arrows indicate unsynapsed sex chromosomes.

Next, we investigated whether BAZ1B regulates DDR signals on meiotic sex chromosome axes. Prior to chromosome-wide spreading of γH2AX to the chromosome-wide domain of the sex chromosomes, DDR signals are established along the unsynapsed axes independent of MDC1 (Ichijima et al., 2011). BRCA1 is a critical factor for the recruitment of the ATR kinase that mediates γH2AX (Royo et al., 2013; Turner et al., 2004), and amplifies DDR signals along the unsynapsed axes for proper establishment of γH2AX domain (Broering et al., 2014). In *Baz1b*cKO, BRCA1, ATR, and its activator, TOPBP1, normally accumulated on unsynapsed axes (Fig. 4A-C). Despite the involvement of BAZ1B in the regulation of γH2AX-MDC1 signaling on ectopic autosomal regions, ectopic signals of BRCA1, ATR, or TOPBP1 was not observed on autosomal regions. Therefore, BAZ1B does not regulate BRCA1, ATR, or TOPBP1 on unsynapsed axes in the early pachytene stage.

### BAZ1B is not required for the formation of pericentric heterochromatin and localization of SMARCA5 during meiosis

**Fig. 5.**
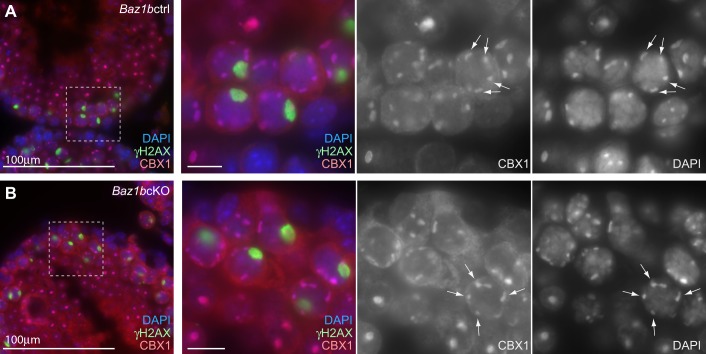
**BAZ1B does not regulate pericentric heterochromatin formation.** Immunostaining of testicular sections stained with (A) CBX1 and (B) γH2AX. Scale bars, 100 µm in the left panels and 10 µm in the right panels. The areas shown with white dotted squares are magnified in the right panels. Arrows indicate autosomal pericentric heterochromatin.

In addition to the role of BAZ1B in DDR signaling, BAZ1B forms a complex with SMARCA5 and regulates chromatin remodeling in somatic cells. Because BAZ1B is associated with the replication of pericentric heterochromatin in somatic cells (Bozhenok et al., 2002), we tested whether *Baz1b* deletion disrupts heterochromatin formation during meiosis. For this analysis, we used paraffin sections of testes that maintain the organization of spermatogenic cells and intact nuclear structures. After the mid-pachytene stage, heterochromatin protein CBX1 intensely accumulated on pericentric heterochromatin in *Baz1b*ctrl (Fig. 5A). This localization was not altered in *Baz1b*cKO (Fig. 5B). Therefore, contrary to its role in somatic cells (Culver-Cochran and Chadwick, 2013), BAZ1B does not regulate localization of CBX1 or pericentric heterochromatin formation during spermatogenesis.

**Fig. 6.**
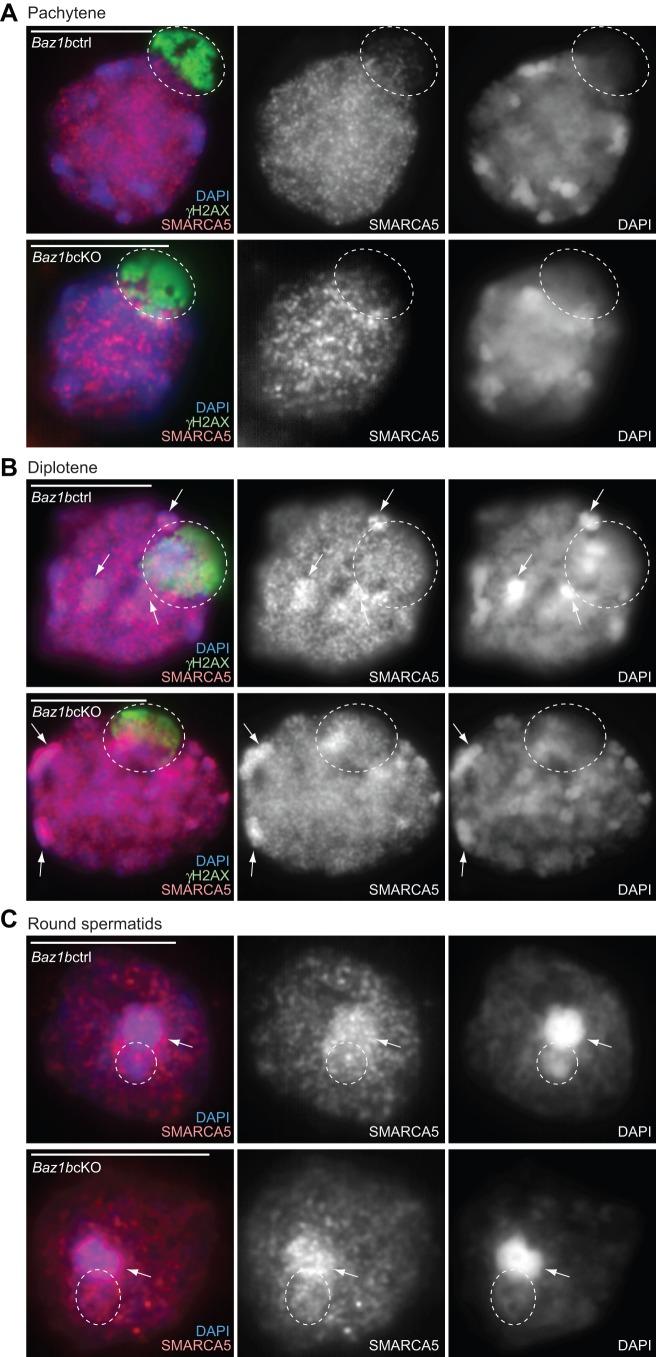
**BAZ1B-independent localization of SMARCA5 in meiotic spermatocytes and in round spermatids.** Immunostaining of slides that preserve the relative 3D chromatin structure of (A) pachytene, (B) diplotene and (C) round spermatid spermatogenic cells (3D slides) stained with SMARCA5 and γH2AX. Single Z-sections were shown. Scale bars, 10 µm. Dotted circles indicate the area of the sex chromosomes. Arrows indicate autosomal pericentric heterochromatin where SMARCA5 accumulates.

We next sought to investigate the localization of SMARCA5 during spermatogenesis and determined whether SMARCA5 localization is affected in *Baz1b*cKO. Because SMARCA5 was found in the γH2AX-containing nucleosomes, we performed double immunostaining with γH2AX and SMARCA5 using slides that preserve the relative chromatin structure of spermatogenic cells (Namekawa, 2014; Namekawa and Lee, 2011). Although SMARCA5 did not localize on the XY body in pachytene spermatocytes (Fig. 6A), SMARCA5 became enriched on the XY body and autosomal pericentric heterochromatin in diplotene spermatocytes (Fig. 6B). Following meiosis in round spermatids, SMARCA5 intensely accumulated on the chromocenter, which is a cluster of pericentric heterochromatin, and also on postmeiotic sex chromatin (PMSC), the heterochromatin domain of the sex chromosomes (Fig. 6C; Namekawa et al., 2006). Localization of SMARCA5 was not affected in *Baz1b*cKO during spermatogenesis (Fig. 6A-C). This result suggests that BAZ1B is not required for the accumulation of SMARCA5 on the sex chromosomes and pericentric heterochromatin.

### BAZ1B-independent regulation of Tyr142 phosphorylation of H2AX during meiosis

**Fig. 7.**
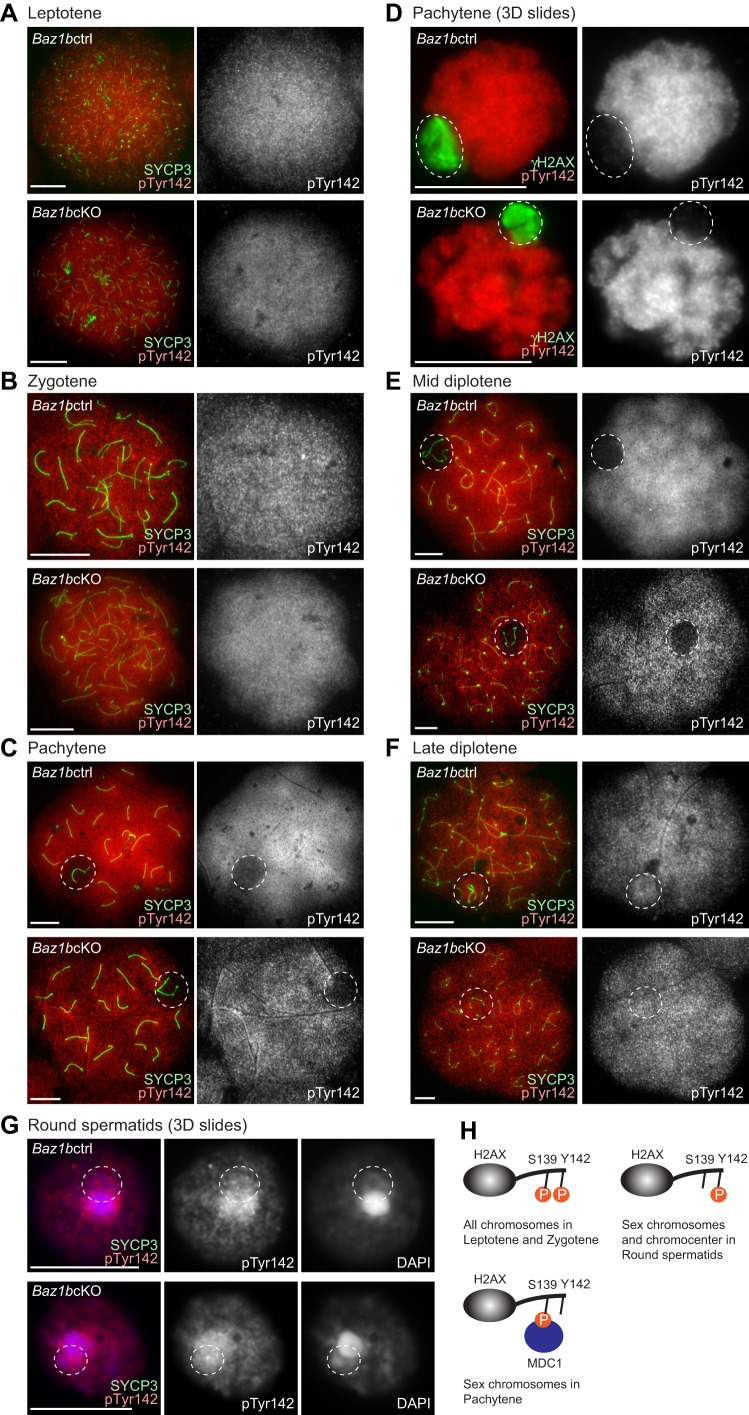
**BAZ1B-independent localization of pTyr142 in meiotic spermatocytes and in round spermatids.** (A-C,D,F) Immunostaining of chromosome spreads of (A) leptotene, (B) zygotene, (C,D) pachytene (E) mid diplotene and (F) late diplotene spermatocytes with pTyr142 and γH2AX. (D,G) Immunostaining of slides that preserve the relative 3D chromatin structure of (D) pachytene and (G) round spermatid spermatogenic cells (3D slides) stained with pTyr142 and γH2AX. Single Z-sections were shown. Scale bars, 10 µm. Dotted circles indicate the area of the sex chromosomes. (H) Models of the mode of two phosphorylation sites of H2AX during meiosis.

Finally, we investigated how the localization of pTyr142 is regulated during critical stages of spermatogenesis when Ser139 adjacent to H2AX is phosphorylated (γH2AX formation). During the leptotene to zygotene stages when γH2AX is detected within the entire nucleus, pTyr142 was detected throughout the nucleus (Fig. 7A,B). However, in pachytene spermatocytes, pTyr142 is excluded from the entire domain of the sex chromosomes (Fig. 7C). The exclusion of pTyr142 was confirmed using slides that preserve the relative chromatin structure of spermatogenic cells (Fig. 7D). These results suggest that dephosphorylation of Tyr142-H2AX occurs specifically on the sex chromosome at the onset of the pachytene stage. The exclusion of pTyr142 was maintained until the mid diplotene stage (Fig. 7E), but pTyr142 reappeared on the sex chromosomes in the late diplotene stage (Fig. 7F). Further, in round spermatids, pTyr142 was detected on both the chromocenter and PMSC (Fig. 7G). Consistent with the results of western blotting, this localization pattern was not affected in *Baz1b*cKO during spermatogenesis (Fig. 7A-G). Thus, phosphorylation of Tyr142 and Ser139 of H2AX are distinctly regulated during spermatogenesis (Fig. 7H). Although both Tyr142 and Ser139 of H2AX can be phosphorylated on autosomes during the leptotene to zygotene stages, Tyr142 was specifically dephosphorylated on the sex chromosomes during the pachytene stage. Furthermore, BAZ1B-independent pTyr142 on the sex chromosomes in late diplotene suggests the possible existence of an unidentified kinase that can mediate pTyr142.

### MDC1 is required for dephosphorylated Tyr142 of H2AX on the sex chromosomes during meiosis

**Fig. 8.**
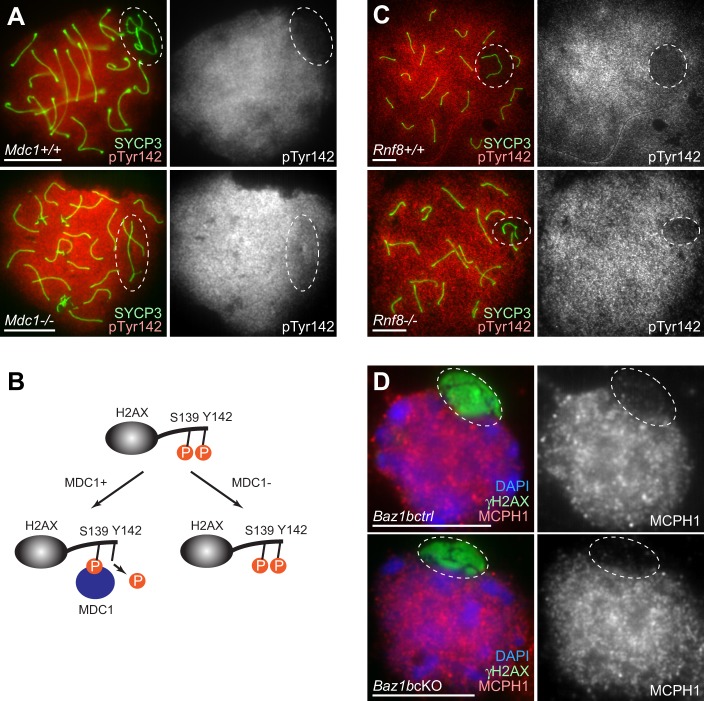
**MDC1, but not RNF8, is required for dephosphorylation of pTyr142 on the sex chromosomes in pachytene spermatocytes.** (A,C) Immunostaining of chromosome spreads with pTyr142 and γH2AX. Scale bars, 10 µm. Dotted circles indicate the area of the sex chromosomes. (B) Models of MDC1-dependent regulation of two phosphorylation sites of H2AX during meiosis. (D) Immunostaining of slides that preserve the relative 3D chromatin structure of spermatogenic cells (3D slides) with MCPH1 and γH2AX. Scale bars, 10 µm. Dotted circles indicate the area of the sex chromosomes.

Next, we next sought to determine the mechanism of the BAZ1B-independent regulation of H2AX Tyr142. There are two waves of γH2AX: one that occurs on the entire nucleus of the leptotene/zygotene stages, and another that occurs on the sex chromosomes during the pachytene stage. MDC1 is specifically required for the later wave, and is dispensable for γH2AX formation in the leptotene/zygotene stages (Ichijima et al., 2011). To determine the regulatory mechanism of H2AX Tyr142, we examined the *Mdc1* knockout (*Mdc1*-KO) mice that are defective in γH2AX spreading on the sex chromosomes during meiosis. In *Mdc1-*KO, dephosphorylation of pTyr142 on the sex chromosomes during meiosis was not observed (Fig. 8A), indicating that MDC1 is required for dephosphorylation of pTyr142 (Fig. 8B). We next investigated whether RNF8 modulates Tyr142 phosphorylation. RNF8 is an essential E3 ubiquitin ligase and a downstream interacting partner of MDC1 in somatic DDR (Huen et al., 2007; Kolas et al., 2007; Mailand et al., 2007). In the male germline, RNF8 mediates ubiquitination of the sex chromosomes and establishes active epigenetic modifications on the sex chromosomes during meiosis, and regulates escape gene activation from inactive sex chromosomes in round spermatids (Sin et al., 2012). Using the *Rnf8* knockout (*Rnf8*-KO) mice, we found that RNF8 is not required for dephosphorylation of pTyr142 on the sex chromosomes during meiosis (Fig. 8C). Therefore, dephosphorylation of pTyr142 is regulated by MDC1 but not by RNF8.

We next examined the meiotic localization of MCPH1 (BRIT1), which recognizes both phosphorylation sites of H2AX (Ser 139 and Tyr 142) in somatic cells (Singh et al., 2012). MCPH1 accumulates on the autosomal regions, but is excluded form the entire domain of the sex chromosomes in wild-type pachytene spermatocytes (Fig. 8D). Additionally, MCPH1 localization is independent of BAZ1B (Fig. 8D) and the localization pattern matches that of pTyr142 during meiosis. These results are consistent with the notions that, in somatic cells, MCPH1 functions in an H2AX-dependent manner but through an MDC1-independent pathway (Wood et al., 2007), and that pTyr142 is incompatible with MDC1 binding (Singh et al., 2012). Thus, these results suggest that MCPH1 is associated with pTyr142 during meiosis.

## DISCUSSION

In this study, we show that BAZ1B is dispensable for fertility as well as major critical events during spermatogenesis, but regulates γH2AX-MDC1 signaling in early pachytene spermatocytes. In spite of the modest distortion of γH2AX-MDC1 signaling on ectopic autosomal regions, which may be due to ectopic meiotic silencing on autosomes rather than the remaining γH2AX signals from unrepaired double-strand breaks, the function of BAZ1B is largely dispensable with regard to other possible functions during spermatogenesis; this is counter to its predicted roles based on BAZ1B's somatic functions. BAZ1B was found to be dispensable for DDR events on unsynapsed axes, pericentric heterochromatin formation, SMARCA5 localization and pTyr142. Since the unique function of BAZ1B was only found in the regulation of γH2AX-MDC1 signaling in the meiotic prophase when extensive DNA damage signaling occurs, BAZ1B is indeed a DNA damage response factor that is involved in the meiotic function, consistent with its function in somatic cells (Xiao et al., 2009); however, BAZ1B is largely dispensable in spermatogenesis and/or could be compensated by other proteins. Further, in spite of the putative complex formation between BAZ1B and SMARCA5, BAZ1B is dispensable for the localization of SMARCA5. It is possible that BAZ1A, a paralog of BAZ1B, could compensate for the function of BAZ1B in spermatogenesis because BAZ1A also localizes to pericentric heterochromatin after the diplotene stage and forms a complex with SMARCA5 in testis lysates (Dowdle et al., 2013). Previous characterization of BAZ1B was largely based on biochemical analyses including purification of protein complexes such as the WICH and WICH-B complexes (Bozhenok et al., 2002; Cavellan et al., 2006). However, to our knowledge, this is the first demonstration of the *in vivo* function of BAZ1B using a loss-of-function mouse model. Our demonstration of the *in vivo* function of BAZ1B is informative with regard to the functional evaluation of the WICH complex, although the existence of the WICH complex during spermatogenesis has not been confirmed yet.

Because (1) the intensity of pTyr142 is maintained in the absence of BAZ1B after mitotic proliferation of gonocytes and spermatogonia, and (2) pTyr142 is established in a BAZ1B-independent manner on the sex chromosomes during the transition between mid to late diplotene stages, it is conceivable that there could be another kinase(s) that mediates pTyr142 in spermatogenesis. However, we could not specify the potential kinase(s) for pTyr142 in this study, or determine whether the kinase function of BAZ1B is compensated for by the potential kinase(s).

Although BAZ1B is dispensable in spermatogenesis, it is notable that SMARCA5 and pTyr142 shared similar localization during meiosis such as exclusion from the XY body in the pachytene stage, recruitment to the XY body in the diplotene stage, and presence on the chromocenter and PMSC in round spermatids. A possible explanation could be that the potential kinase(s) for pTyr142 could form a complex with SMARCA5 and mediate pTyr142 in the absence of BAZ1B.

Our results reveal that MDC1 is required for dephosphorylation of pTyr142. Because MDC1 directly binds to the adjacent pSer139 of H2AX (γH2AX), the dephosphorylation of pTyr142 could be directly coupled with MDC1. The EYA family of proteins was reported to be phosphatases of pTyr142 (Cook et al., 2009; Krishnan et al., 2009); it is possible that one of the four EYA proteins (EYA1, EYA2, EYA3, and EYA4) may be involved in this process. However, EYA proteins are functionally redundant (Tadjuidje and Hegde, 2013) and it is difficult to specify the enzyme that mediates dephosphorylation of pTyr142 on the sex chromosomes during meiosis. Our unpublished data suggest that EYA3 is not essential in this process (T.J.B., R.S.H., Richard A. Lang and S.H.N., unpublished), but it is possible that other EYA proteins could compensate for EYA3. According to our previous microarray data (Namekawa et al., 2006), EYA3 and EYA4 are highly expressed during spermatogenesis. Therefore, EYA4 could compensate for the function of EYA3 during spermatogenesis.

Additionally, our analyses reveal that two waves of γH2AX formation during meiosis are distinctly regulated in terms of two adjacent phosphorylation sites (Ser139 and Tyr142) of H2AX. Consistent with this distinct regulation, MDC1 is only required for the spreading of γH2AX on the sex chromosomes in the pachytene stage, but not for nuclear γH2AX in the leptotene and zygotene stages (Ichijima et al., 2011). These results further support the notion that pTyr142 is a switch for DDR regulation (Cook et al., 2009) and could also be involved in the regulation of sex chromosomes during meiosis. Intriguingly, pTyr142 persists on PMSC in round spermatids after the disappearance of γH2A. It would be promising to investigate the function of pTyr142 during meiosis and its functional interaction with γH2AX.

## MATERIALS AND METHODS

### Animals

All animal work was conducted in accordance with national and institutional guidelines. *Baz1b^tm2a(KOMP)Wtsi^* was generated as part of the International Knockout Mouse Consortium program at the Wellcome Trust Sanger Institute and obtained from the National Institutes of Health (NIH)-sponsored Knockout Mouse Program (KOMP) Repository located at the University of California Davis. Mice with the floxed allele of *Baz1b* exon 5 were generated after the removal of selection cassette crossing with the ROSA26Flpo mice from the Jackson Laboratory. *Ddx4-cre* transgenic mice (Gallardo et al., 2007) were obtained from the Jackson Laboratory. Because the *Ddx4-cre* allele needs to be transmitted from the paternal allele to generate mice with germline-specific conditional deletions, males with *Baz1b^+^/ΔDdx4-cre* (6 weeks up to 18 months old) were mated with females homozygous for the floxed allele of *Baz1b* exon 5 (*Baz1b*F/F) (4 weeks up to 12 months old), and the conditional deletion model *Baz1b F/ΔDdx4-cre* (*Baz1b*cKO) was obtained. Littermates of *Baz1b F/^+^Ddx4-cre* were used as controls (*Baz1b*ctrl). Heterozygous for *Baz1b*^+/−^ males (6 weeks old up to 4 months old) and *Baz1b*^+/−^ females (6 weeks old up to 6 months old) were mated. However, only one homozygous male (*Baz1b*^−/−^) was obtained in two total years of breeding. These mice are on a mixed background. *Mdc1*-KO, *Rnf8*-KO mice are on C57BL/6 backgrounds and were previously reported (Lou et al., 2006; Minter-Dykhouse et al., 2008).

### Antibodies

The antibody list used in this study is available in supplementary material Table S1. Briefly, two independent anti-pTyr142 antibodies are used for western blotting in Fig. 1D: the antibody from Millipore was used for the left panel, and the antibody from Abcam was used for the left panel. Anti-pTyr142 immunofluorescence (Figs 7,8) was performed with the antibody from Abcam.

### Western blotting

Single cell suspensions were prepared from whole testes. After the removal of tunica albuginea, 0.5 mg/ml collagenase and 16 µg/ml DNase I in PBS was added to PBS to create a total volume of 5 ml. Cells were incubated at 32°C for 15 min. Clumps were loosened by pipetting for 1 min, followed by centrifugation at 1000 rpm at RT for 5 min. After washing with PBS, cells were resuspended in 10 ml PBS and counted. Cells were resuspended in sample buffer at a concentration of 4×10^6^ cells/100 µl and lysed by sonication. For western blotting analysis, the cell lysates (corresponding to 4×10^5^ cells per well) were loaded in 8% (for BAZ1B) or 12% (for H2AX) acrylamide gels, and then separated through electrophoresis. After transfer, the blots were incubated with specific antibodies, including BAZ1B and pTyr142, as well as α-tubulin and total H2AX, which were used as internal controls. Following incubation with horseradish peroxidase (HRP)-conjugated secondary antibodies, chemiluminescence signals were detected by exposure to X-ray films.

### Spermatogenesis slide preparation

Analysis of sex chromosomes during meiosis was performed using hypotonic treatment as described (Peters et al., 1997). In order to conserve the morphology of meiotic chromatin and the relative three-dimensional nuclear structure in mouse testes, analysis of sex chromosomes in round spermatids was preformed using slides that were prepared as described (Namekawa, 2014; Namekawa and Lee, 2011; Namekawa et al., 2006). For these slide preparations, mutants and littermate controls were processed at between 40 to 130 days of age postpartum.

### Immunofluorescence microscopy of spermatogenesis slides and data analysis

Slides were incubated in PBT (0.15% BSA, 0.1% Tween 20 in PBS) for 60 min prior to overnight incubation at room temperature with the antibodies listed in supplementary material Table S1. Thereafter, slides were washed 3 times for 5 min each in PBS with 0.1% Tween 20, incubated with secondary antibodies (Invitrogen or Jackson ImmunoResearch) at 1:500 for 60 min in PBT, washed in PBS plus 0.1% Tween 20, and mounted in Vectashield with DAPI. Images of germ cells were acquired with an ECLIPSE Ti-E microscope (Nikon) and Zyla 5.5 sCMOS camera (Andor Technology), with 20×, 60×, and 100× CFI Apochromat TIRF oil immersion lenses (Nikon), numerical aperture 1.40; image acquisition was performed using NIS-Elements Basic Research software (Nikon). Images were taken at RT (∼22°C). Phylum, Volocity 3D Image Analysis (PerkinElmer), NIS-Elements Basic Research, and NIS-Elements Viewer (Nikon) were used for image analysis. Photoshop and Illustrator (Adobe) were used for composing figures. Particular stages of primary spermatocytes were determined by staining for H2AX, SYCP3 and/or H1T. For data analysis, the matched substage of meiosis was analyzed in controls and mutants. All data were confirmed with at least two or three independent mice. Total numbers of analyzed nuclei in at least two independent experiments are shown in each panel.

### Histology, immunohistochemistry, TUNEL staining and diameter analyses

For preparation of testicular paraffin blocks, testes without tunica albuginea were fixed with 4% paraformaldehyde (PFA) overnight. Testes were dehydrated and embedded in paraffin. For immunohistochemistry, paraffin sections 6 μm thick were deparaffinized and autoclaved in Target Retrieval Solution (DAKO) at 121°C for 10 min. After incubation with blocking solution (0.15% bovine serum albumin, 0.1% Tween-20 in PBS) for 1 h at room temperature, sections were incubated with antibodies at 4°C overnight. The resulting signals were detected by incubation with Alexa488- or Alexa594-conjugated secondary antibody (Molecular Probes). Sections were counterstained with DAPI and images were processed with Adobe Photoshop. For TUNEL staining, sections were deparaffinized and then treated with 15 μg/ml Proteinase K for 10 min at 37°C and stained with In Situ Cell Death Detection Kit (Roche) following the manufacturer's protocol. The sections were counterstained with DAPI and analyzed. Seminiferous tubule diameters were determined using NIS-Elements Basic Research software (Nikon). Using the software, the circumferences of the tubules were manually traced, the total area of each tubule was then recorded into Microsoft Excel and converted into the diameter using the equation: 
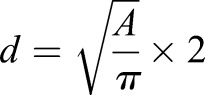
 (d: diameter of a tubule; A: area of a tubule). Only cross sections that appeared circular in shape were analyzed.

## Supplementary Material

Supplementary Material
