# Supplementary material for: BAZ1B is dispensable for H2AX phosphorylation on Tyrosine 142 during spermatogenesis
Source: Biol Open. 2015 May 15;4(7):873–84. doi: 10.1242/bio.011734 (PMC4571090; doi:10.1242/bio.011734)
Supplement: Supplementary Material [file supp_4_7_873__index.html]

BAZ1B is dispensable for H2AX phosphorylation on Tyrosine 142 during spermatogenesis — BAZ1B is dispensable for H2AX phosphorylation on Tyrosine 142 during spermatogenesis — Supplementary Material 

# BAZ1B is dispensable for H2AX phosphorylation on Tyrosine 142 during spermatogenesis

## BIO011734 Supplementary Material

- Supplementary Material
