# Supplementary material for: BAZ1B is dispensable for H2AX phosphorylation on Tyrosine 142 during spermatogenesis
Source: Biol Open. 2015 May 15;4(7):873–84. doi: 10.1242/bio.011734 (PMC4571090; doi:10.1242/bio.011734)
Supplement: Supplementary Material [file supp_bio.011734_BIO011734supp.pdf]

Table S1. Antibody list

Name	Origin	Product #	Applications
Rabbit anti-H2AX	Cell Signaling	7631	WB
Mouse anti-γH2AX	Millipore	05-636	IC, IHC, IP
Rabbit anti-BAZ1B	Abcam	ab50632	WB
Rabbit anti-H2AX (phospho Y142)	Abcam	ab94602	IC, WB
Rabbit anti-H2AX (phospho Y142)	Millipore	07-1590	WB
Mouse anti-α-Tublin	Sigma	9026	WB
Mouse anti-SYCP3	Abcam	ab97672	IC
Rabbit anti-SYCP3	Novus	NB300-232	IC
Rabbit anti-ATR	Cell Signaling	2790	IC
Rabbit anti-TOPBP1	Gift from Junjie Chen		IC
Rabbit anti-BRCA1	Generated by Namekawa lab		IC
Guinea Pig anti-H1T	Gift from Mary Ann Handel		IC, IHC
Rabbit anti-Cleaved Caspase 3	Cell Signaling	9661	IHC
Sheep anti-MDC1	ABD Serotec	AHP799	IC
Rabbit anti-CBX1	Abcam	ab10478	IHC
Rabbit anti-SMARCA5	Abcam	ab3749	IC
Rabbit anti-MCPH1	Bioss	bs-11227R	IC
